# Effects of Thermal Stress on the Formation and Cracking Behavior of Nickel-Based Superalloys by Selective Laser Melting Based on a Coupled Thermo-Mechanical Model

**DOI:** 10.3390/ma15248968

**Published:** 2022-12-15

**Authors:** Shijin Nie, Lin Li, Qin Wang, Rongxia Zhao, Xin Lin, Furong Liu

**Affiliations:** 1 Key Laboratory of Trans-Scale Laser Manufacturing, Beijing University of Technology, Ministry of Education, Beijing 100124, China; 2 Institute of Laser Engineering, Faculty of Materials and Manufacturing, Beijing University of Technology, Beijing 100124, China; 3 State Key Laboratory of Solidification Processing, Northwestern Polytechnical University, Xi’an 710072, China

**Keywords:** selective laser melting, thermal behavior, stress field, cracking

## Abstract

Complex thermal cycles and stress fields commonly occur in the selective laser melting process for nickel-based superalloys, which are prone to generating cracks and decreasing the performance of forming parts. In this paper, the reasons for cracking were analyzed by combining the experiment with the evolution behavior of the temperature field/stress field during the solidification process of a nickel-based superalloy (FGH96) via a three-dimensional finite element thermo-mechanical coupling model. It showed that a radial temperature distribution of the melting pool led to a similar distributed stress; as a result, the value declined slowly along the scanning direction but declined quickly along the direction perpendicular to the scanning direction. A stress concentration with maximum stress up to 339 MPa was found at the center of the molten pool, easily causing a crack in SLM. It was found that both the initiation and propagation of the cracks were along the grain growth direction and were affected by the epitaxial growth of columnar crystals. For the case of process parameters with relatively high power or low scanning speed, the stress value of the molten pool during solidification was more than 370 MPa so as to form a large area of cracks. The adjustment of the rotation angle between the adjacent layers was effective at avoiding stress accumulation in the building direction and prevent the formation of long grain boundaries, thus avoiding crack propagation. The present study lays a foundation for the wide applications of selective laser melting technologies in nickel-based superalloys.

## 1. Introduction

With the development of manufacturing technology, the process requirements of existing nickel-based superalloy devices tend to be more integrated, complex, precise, and thin-walled. The traditional nickel-based superalloy manufacturing process is gradually failing to meet the requirements of the existing process [1,2,3]. The “Laser Additive Manufacturing” technology, which has emerged in recent decades, has attracted more and more attention [4]. It is an integrated manufacturing method that adds materials layer by layer in a continuous piling up. It can achieve precision manufacturing for some complex structural parts [5] (truss structure [6], ultra-thin grid [7], etc.). Selective laser melting (SLM) technology has a large temperature gradient and cooling speed [8]. At the same time, because nickel-based superalloys have a high melting point and are easy to crack, it is challenging to use SLM to form them. Consequently, it is crucial to investigate the evolution laws of the temperature field/stress field and its effect on the formation of a crack.

At present, in the research on the stress fields of the laser additive manufacturing of nickel-based superalloys, Mercelis et al. [9] explained the generation mechanism of residual stress by temperature gradients. Later, research on process parameters mainly focused on the influence of scanning strategies to process thermal behavior and stress [10,11,12,13]. The scanning strategy was found to play a significant role in stress distribution, which is anisotropic and has higher thermal stress along the direction of laser scanning. Roberts et al. [14] analyzed in greater detail the effects of process parameters, the scanway length, preheating temperature, and substrate characteristics on residual stress. Along with the effect of process parameters, other researchers also looked at where the overall concentration of residual stress was in the model on a larger scale. Other studies indicated that short distance scanning can effectively reduce stress [15,16]. Denlinger et al. [17] and Li et al. [18] investigated the stress distribution in the multi-layer and multi-pass forming process of superalloys, and their research demonstrated that the stress gradually increased along the deposition height during deposition. Vrancken et al. [19] analyzed the residual stress of the parts produced by SLM, and the results demonstrated that residual stresses significantly impacted the anisotropy of forming.

In addition to the influence of process parameters and building height on the stress field, other studies on thermodynamic coupling attribute cracking to stress [20]. Li et al. [21] studied the stress and crack distribution at different positions in the process of multi-channel scanning, but did not thoroughly study the relationship between them. Marcel et al. [22] discovered that decreasing the diameter of the spot increased the depth of fusion, which in turn reduced the number of cracks by remelting the cracks. The research of Gao et al. [23] demonstrated that the process parameters affect the structure and cracking sensitivity of materials by affecting the cooling rate. Sun et al. [24] found that increasing the scanning speed and scanning distance could reduce cracking sensitivity. It is evident from the studies on the thermal coupling that the effect of temperature field/stress field on cracking during solidification was not thoroughly investigated.

In this study, numerical simulation and experimental validation are merged to reveal the evolution law of the temperature field/stress field of nickel-based superalloys during SLM and to investigate the mechanism of crack formation and propagation. On the basis of the heat conduction theory of continuous mediums, a three-dimensional finite element thermal-mechanical coupling model was constructed, and the temperature field/stress field information, including the change in the temperature gradient and equivalent stress at different locations of the molten pool, were analyzed quantitatively. During solidification, the temperature distribution in the longitudinal section of the molten pool determined the radiant distribution of stress. Experimental results were compared to the simulation results. From the evolution of the temperature and stress fields, the cause of grain boundary cracking at large angles was determined. This lays the groundwork for the widespread application of selective laser melting technology in nickel-based superalloys.

## 2. Experimental and Computational Procedures

### 2.1. Experiment

**Figure 1:**
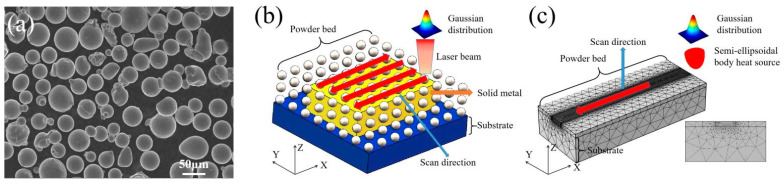
(**a**) SEM image of FGH96 powders; (**b**) Schematic diagram of selective laser melting; (**c**) Construction of the 3D finite element model.

Nickel-based superalloy powders of FGH96 were selected for the experiment, which have a high Ti/Al content, as shown in Table 1. FGH96 has rich elements, including Cr, Co, Mo, Ti, Al, etc., with a complex microstructure evolution that takes place during the solidification process, which has a certain representative in the nickel-base superalloy. Therefore, FGH96 was used as the research material in this study. The powders made using gas atomization had a good spherical shape and a size ranging from 15 to 53 μm, as shown in Figure 1a. SLM is an integrated manufacturing technology that uses a high-energy laser beam to selectively melt the metal powder and stack layer by layer. The XY plane is the laser scanning plane and Z is the building direction, as shown in Figure 1b. The SLM process was performed based on the EP-M260 machine. A reciprocating scanning scheme was used in the experiment. The process parameters of the experiment are shown in Table 2. After the SLM process, metallographic samples were ground, polished, and etched using a mixed chemical reagent of 7 mL HCl, 7 mL C_2_H_5_OH and 1.4 g CuSo_4_ liquids. The microstructure was then observed using an optical microscope (OM) and scanning electron microscope (SEM). The crystal orientation analysis was also performed using electron backscatter diffraction (EBSD). 

### 2.2. Computational Procedures

In order to predict the evolution of the temperature field/stress field in SLM, in this paper, a three-dimensional finite element model was established based on the heat conduction theory of continuum. As shown in Figure 1c, the geometry of the established model was composed of three regions, namely the substrate, powder bed, and forming area. The size of the substrate area was 1500 μm × 600 μm × 260 μm, while that of the powder bed thickness was 40 μm. To save calculation time, non-uniform mesh division was carried out, as shown in Figure 1c. In the process of model construction, a free tetrahedral mesh was used. The suitable mesh sizes of the laser scanning area of the powder layer (12 μm) and substrate (52 μm) were obtained, respectively, using a convergence method of automatic control in the COMSOL Multiphysics software. 

The evolution of the temperature field in the selective laser melting process is usually described by the Fourier equation [25] and is expressed as:

(1) $$\rho c\frac{\partial T}{\partial t}=\frac{\partial }{\partial x}\left(k_{x}\frac{\partial T}{\partial x}\right)+\frac{\partial }{\partial y}\left(k_{y}\frac{\partial T}{\partial y}\right)+\frac{\partial }{\partial z}\left(k_{z}\frac{\partial T}{\partial z}\right)+Q\left(x,y,z,t\right)$$

where ρ, C are the density and specific heat capacity. k_x_, k_y,_ and k_z_ are the spatial three thermal conductivity in the spatial coordinate system. T and t are temperature and time, respectively. Q is heat generation and heat per unit volume obtained by laser radiance. The penetration characteristics of laser radiation in the powder bed and the laser with Gaussian distribution in the experiment were considered; a semi-ellipsoidal Gaussian body heat source is, therefore, used as below [26]:

(2) $$I\left(x,y,z\right)=q_{0}\cdot exp\left[-2\left(\frac{x^{2}}{a^{2}}+\frac{y^{2}}{b^{2}}+\frac{z^{2}}{c^{2}}\right)\right]$$

where q_0_ is the coefficient derived from the conservation of energy, given as:

(3) $$q_{0}=\frac{2^{5/2}\cdot \beta \cdot P}{\pi^{3/2}\cdot a\cdot b\cdot c}$$

where x, y, z are position coordinates; a, b, c are ellipsoidal semiaxis; β represents the laser absorption rate of the material; P represents the laser power input to the powder bed.

The boundary conditions, including convection and radiation, were considered in the model as follows:

(4) $$q_{c}=-h_{c}\left(T_{1}-T_{0}\right)$$

(5) $$q_{r}=-\omega \varphi \left(T_{1}^{4}-T_{0}^{4}\right)$$

where q_c_ and q_r_ represent the heat flux of convection and radiation. ω is the radiation coefficient (0.8), φ is the Stephen–Boltzmann constant (5.67 × 10^−8^ W/(m^2^·K^4^)), h_c_ is the convective coefficient (80 W/(m^2^·K)), T_1_ is the material temperature, and T_0_ is the substrate temperature (80 °C).

The phase change was also considered in the simulation, and the enthalpy changes of the material, represented by heat capacity, can also be described below [27]:

(6) $$C_{p}=\{\begin{array}{c} C_{p,sensible}\;for\;T<T_{m}-0.5\Delta T_{m}\;or\;T>T_{m}+0.5\Delta T_{m}\\ C_{p,modified}=C_{p,sensible}+H/\Delta T_{m}\;for\;T_{m}-0.5\Delta T_{m}<T<T_{m}+0.5\Delta T_{m} \end{array}$$

where H is the latent heat of phase transformation, ΔT_m_ is the melting range, C_p_ is the heat capacity, and T_m_ is the melting temperature (1553 K) [28].

Assuming FGH96 is an isotropic material, the stress–strain relationship in the Cartesian coordinate system can be expressed as [29]:

(7) $$\varepsilon_{x}=\frac{1}{E}\left[\sigma_{x}\mu \left(\sigma_{y}+\sigma_{z}\right)+\alpha \Delta T\right]$$

(8) $$\varepsilon_{y}=\frac{1}{E}\left[\sigma_{y}\mu \left(\sigma_{x}+\sigma_{z}\right)+\alpha \Delta T\right]$$

(9) $$\varepsilon_{z}=\frac{1}{E}\left[\sigma_{z}\mu \left(\sigma_{x}+\sigma_{y}\right)+\alpha \Delta T\right]$$

where E is the elastic modulus and α is the thermal expansion coefficient.

In the process of establishing the finite element model in this paper, the Von Mises yield criterion was used to analyze the deformation of the materials. The formula of the equivalent force is:

(10) $$\sigma =\frac{\sqrt{2}}{2}\sqrt{{\left(\sigma_{1}-\sigma_{2}\right)}^{2}+{\left(\sigma_{2}-\sigma_{3}\right)}^{2}+{\left(\sigma_{3}-\sigma_{1}\right)}^{2}}$$

The formula of the equivalent strain is:

(11) $$\varepsilon =\frac{\sqrt{2}}{2\left(1+\mu \right)}\sqrt{{\left(\varepsilon_{1}-\varepsilon_{2}\right)}^{2}+{\left(\varepsilon_{2}-\varepsilon_{3}\right)}^{2}+{\left(\varepsilon_{3}-\varepsilon_{1}\right)}^{2}}$$

where σ_1_, σ_2_, σ_3_ are the principal stress in three directions and ε_1_, ε_2_, ε_3_ are the principal strain in three directions. μ is Poisson’s ratio.

## 3. Results and Discussion

### 3.1. Experiment Results

#### 3.1.1. Experiment Results of Different Process Parameters

**Figure 2:**
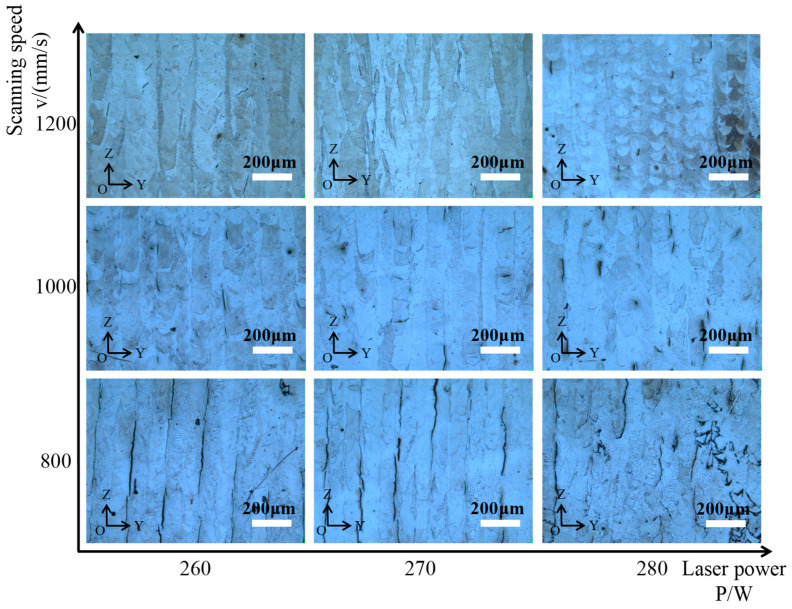
Sample YOZ cross-section under different process parameters.

Figure 2 shows the macroscopic morphology characteristics of the samples under different process parameters. It can be seen from the figure that the influence of the scanning speed on the number of cracks is greater than that of the power. Under the same laser power, with the scanning speed increased from 800 mm/s to 1200 mm/s, the number and length of the cracks decreased. At the same time, incomplete fusion holes gradually appeared. In addition, under the same scanning speed and increased laser power from 260 W to 280 W, the remelting of the latter layer on the previous layer accumulated, and the length of the cracks gradually became shorter while the number of cracks gradually decreased. This is consistent with the research results of others [22]. However, when the laser power increased to 280 W, the heat input exceeded the requirement of crack fusion, which increased the temperature gradient and the internal stress between the layers leading to the emergence of a large number of cracks again. 

**Figure 3:**
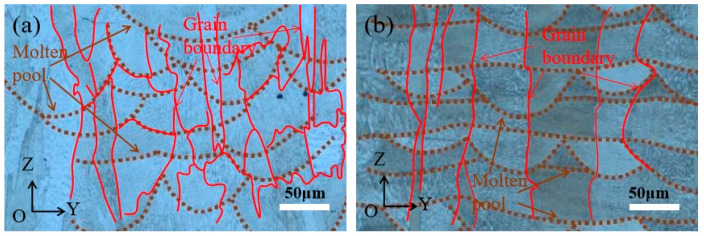
Molten pool morphology of YOZ surface of samples prepared at different rotation angles between adjacent layers. (**a**) 67°; (**b**) 90°.

Figure 3 shows the molten pool morphology of the XOZ plane of samples prepared at rotation angles of 67° and 90° between the adjacent layers. At the interlayer rotation angle of 67°, the molten pool trajectories between the adjacent layers were not obviously regular, which led to the preferential growth of dendrites with different orientations, resulting in irregular columnar or equiaxed grain structures. The disorderly grain boundaries also inhibited the propagation of microcracks. At an interlayer angle of 90°, the molten pool trajectories of the two adjacent layers were perpendicular to each other, and the subsequent thermal cycling process of layer-by-layer scanning produced a relatively vertical and thick columnar grain structure, which also provided a continuous channel for the initiation and propagation of microcracks, making it easy to form long and straight cracks.

#### 3.1.2. Crack Morphology

**Figure 4:**
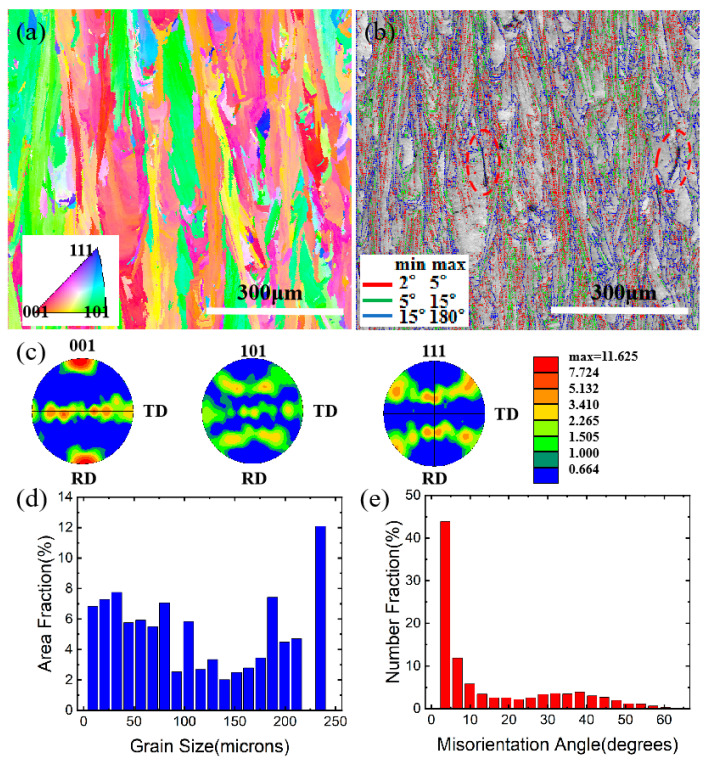
EBSD analyses. (**a**) grain morphology; (**b**) grain boundary angle distribution; (**c**) inverse pole figure; (**d**) grain size statistical diagram; (**e**) grain boundary angle statistical diagram.

Figure 4 shows the microstructure variation with the EBSD analyses. It can be seen that most of the crystals are columnar and are mainly along the building direction. The growth of the columnar crystals is mainly along the <001> direction. More than 50% of the columnar crystals are longer than 80 μm, with the largest reaching 240 μm, as shown in Figure 4d. Figure 4e shows that most of the grain boundaries belong to the small angle boundaries and are mainly located between 3 and 6 degrees, except for a few large angle boundaries over 50 degrees. Additionally, cracks are also found along the large angle grain boundary, as seen in the red dashed circle in Figure 4b.

**Figure 5:**
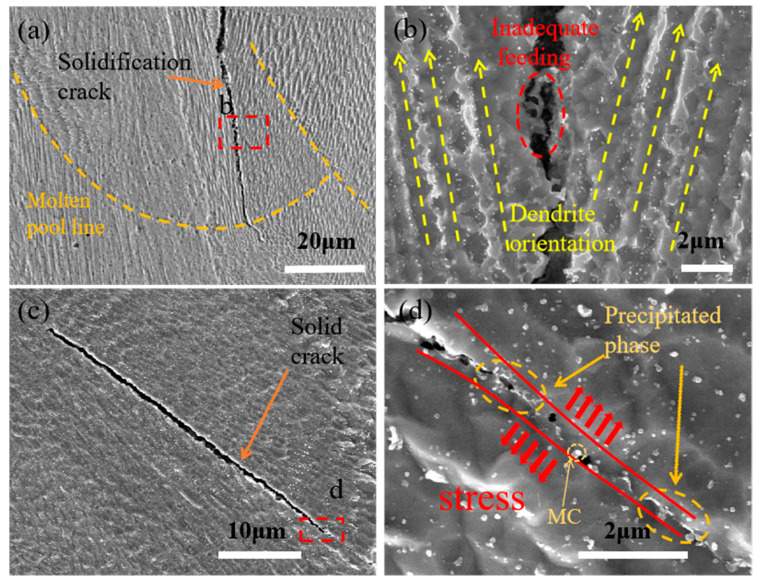
(**a**) Solidification crack; (**b**) Local amplification of the area inside the dotted box in (**a**); (**c**) Solid crack; (**d**) Local amplification of (**c**).

Figure 5a shows a typical solidification crack. Solidification cracks are generated in the solidification process of the molten pool, so there is nothing in common between the morphology of both sides of the cracks. It can be seen from Figure 5a that the crack originates in the cellular crystal zone, that is, in the central area of the molten pool in the previous layer. During the subsequent solidification process, the molten metal fed the cracks to a certain extent, but the accompanying thermal stress continued to act on both sides of the cracks, resulting in insufficient feeding and expanding of the cracks. At the same time, some solidified molten metal can be left in the cracks, as shown in Figure 5b.

Figure 5c shows a typical solid crack. This is due to the continuous thermal influence of the forming area during the manufacturing process, which leads to stress concentration and directly tears the weak parts of the forming area. It can be seen from Figure 5d that there are some precipitates and holes around the crack source. Many studies [30,31,32] considered that the cracking of nickel-based superalloys was caused by liquefaction cracking due to the presence of the low melting point phase. A solid crack is a kind of crack formed in the solid region after complete solidification. Therefore, the shapes on both sides of the crack coincide to a high degree, and there are no impurities in the crack. As shown in Figure 5d, cracks occur along the grain boundaries under tensile stress. Remarkably, it was found that in addition to liquefaction cracks, solid cracks and solidification cracks exist simultaneously in the forming region, and all kinds of cracks do not exist alone but have a certain relationship. This is consistent with the experimental phenomenon observed in reference [33].

### 3.2. Simulation Results

#### 3.2.1. Model Verification

**Figure 6:**
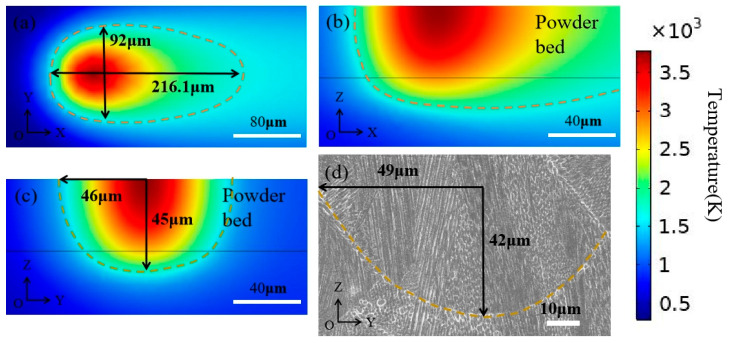
Model verification. (**a**) Temperature field simulation results of the XOY section; (**b**) Temperature field simulation results of the XOZ section; (**c**) Temperature field simulation results of the YOZ section; (**d**) Morphology of the molten pool in the experiment.

Figure 6 depicts the molten pool morphology when the selective laser melting parameters are 270 W and 1200 mm/s. For the FGH96, its melting point is about 1553 K. The length, and width of the molten pool are the longest melting lines on the *x* and *y* axes, respectively; the depth of the molten pool is the melting line from the surface of the powder layer to the bottom of the molten pool along the *z*-axis. Figure 6a,b shows the profiles of the molten pool XOY and XOZ. The length of the molten pool is 216 μm and the width is 92 μm; Figure 6c shows the molten pool profile on the YOZ surface of the molten pool and the depth of the molten pool, which is 45 μm. Figure 6d shows the experimental results of the longitudinal section of the molten pool under the same process parameters. The width of the molten pool is 98 μm, and the depth is 42 μm. Therefore, although there is a certain error between the simulation and the experimental results, it is within the acceptable range. The accuracy of this model is verified.

#### 3.2.2. Quantitative Thermal Behavior within the Molten Pool

**Figure 7:**
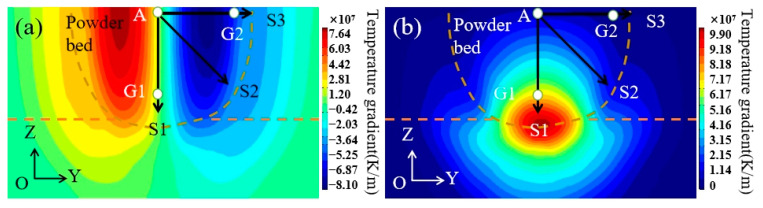
Simulated temperature gradients along the (**a**) Y direction for the melting pool (Gy); (**b**) Z direction for the melting pool (Gz).

**Figure 8:**
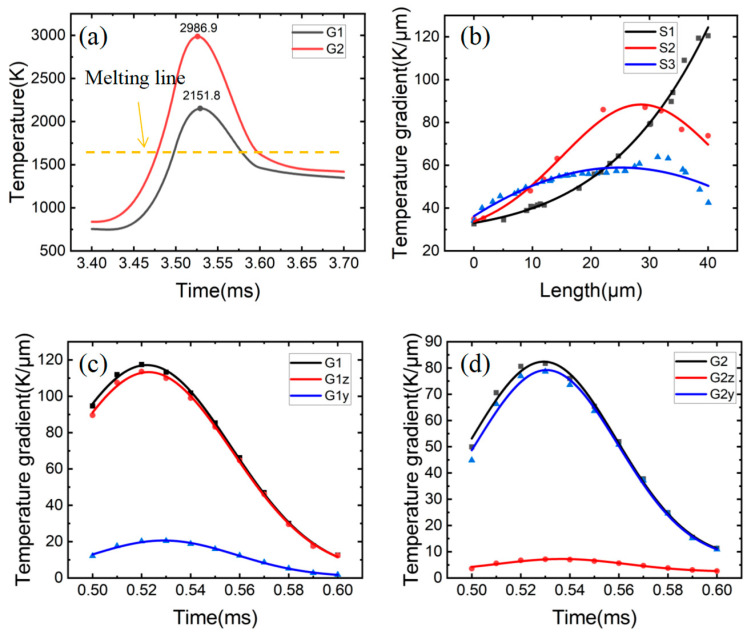
Model verification. (**a**) Temperature change at different positions of molten pool; (**b**) Map of temperature gradient with location; (**c**) Temperature gradient at the bottom of molten pool; (**d**) Temperature gradient on both sides of molten pool.

Figure 7 shows the distributions of the temperature gradient within the melting pool along the Y and Z directions. The temperature gradient at the bottom of the molten pool in the Gz direction was the largest, and the farther away from the bottom of the molten pool, the smaller the temperature gradient in the Gz direction became. In the Gy direction, the temperature gradient on both sides of the molten pool was the largest and had a certain symmetry. In order to study the evolution law of the temperature field in different positions of the molten pool, the temperature and temperature gradient changes of G1 and G2 points in the molten pool were selected, as shown in Figure 8a,c,d. The temperature gradient changes in the different directions from point A in the center of the molten pool to S1, S2, and S3 are shown in Figure 8b.

Figure 8b shows the evolution of the temperature gradient at different positions of the molten pool, the position of which can be seen as the temperature gradient at the center line of the molten pool increases from 3.2 × 10^7^ K/m to 1.2 × 10^8^ K/m and further away from point A. Because the temperature gradient along the Z direction and at the center line is dominant, reaching the maximum at the bottom of the molten pool, the temperature gradient at both sides of the molten pool first increases to 8.7 × 10^7^ K/m and then decreases with the distance from point A. This is because the temperature gradient along the Y direction on both sides of the molten pool plays a dominant role, reaching the peak value of the temperature gradient along the Y direction first and then decreasing.

In the G2 point, a temperature gradient of about 8 × 10^7^ K/m is formed along the Y direction, which is much greater than G2z, and G2y plays a leading role, as shown in Figure 8d. At the bottom of the molten pool (G1), there was a huge temperature gradient (about 1.2 × 10^8^ K/m) along the Z direction, which was much larger than that of G1y and played a dominant role. It is easy to meet the dendrite growth conditions, resulting in the rapid epitaxial growth of dendrites along the Z direction, as shown in Figure 8c. From the temperature curve in Figure 8a, it can be seen that the temperature is the lowest, and the corresponding solidification time is the shortest at the bottom of the molten pool.

#### 3.2.3. Stress Field Information within the Molten Pool

**Figure 9:**
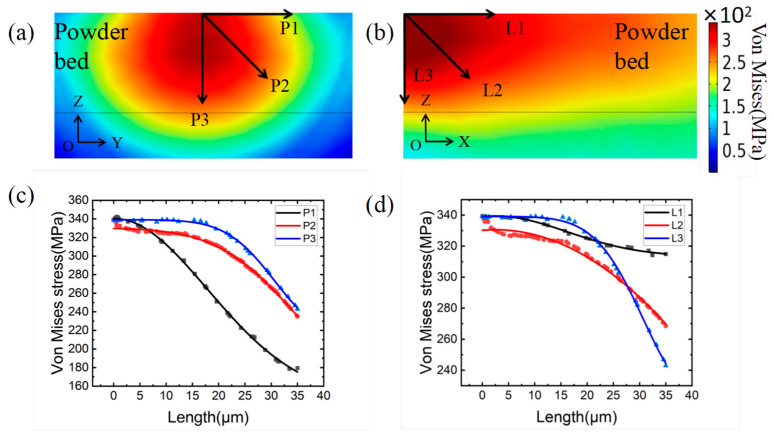
(**a**) Simulation results of YOZ section stress field; (**b**) Simulation results of XOZ section stress field; (**c**) Stress value distribution in different paths of YOZ section; (**d**) Stress value distribution in different paths of XOZ section.

The stress field of the molten pool in the YOZ section is shown in Figure 9a. It can be seen that the stress value near the center of the molten pool is the highest and that the value decreases as one moves away from the center. The stress value varies in three directions, which is shown in Figure 9c: from 339 MPa to 173 MPa in the P1 (Y) direction, from 339 MPa to 245 MPa in the P3 (Z) direction, and from 339 MPa to 238 MPa in the P2 direction. The stress field of the molten pool in the XOZ section is depicted in Figure 9b. It can be concluded that the stress value at the front end of the molten pool is the highest and that the stress value decreases with the distance from the center of the molten pool. Correspondingly, the stress value of the molten pool decays the slowest at the tail of the pool (X direction). It is due to the residual temperature in the tail of the molten pool that results in thermal expansion. On both sides of the molten pool (Y direction), the reduction in stress is the quickest. During solidification, the temperature distribution in the longitudinal section of the molten pool determines the radiant distribution of stress. 

### 3.3. Effect of the Process Parameter of SLM

**Figure 10:**
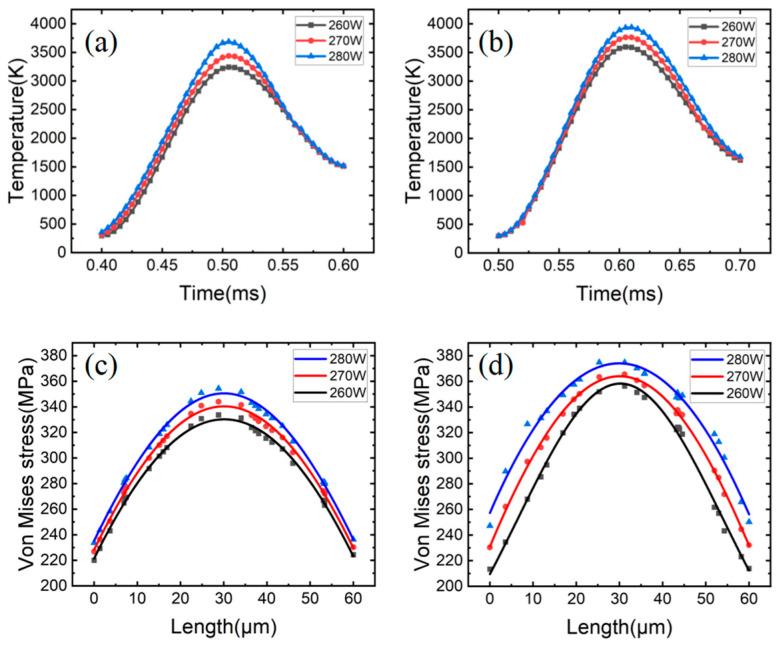
Temperature variation under different process parameters (**a**) 1200 mm/s; (**b**) 1000 mm/s. Change in stress value under different process parameters (**c**) 1200 mm/s; (**d**) 1000 mm/s.

Figure 10a,b shows the temperature change at the center of the molten pool under different process parameters. It is obvious that the temperature change trend is consistent regardless of the power or scanning speed. During the selective laser melting process, when the laser power remains constant and the scanning speed increases to 200 mm/s, the maximum temperature of the molten pool decreases by approximately 250 K. With a 10 W increase in laser power, the maximum temperature of the molten pool increases by approximately 170 K at a constant scanning speed.

Figure 10c,d exhibits the variation in the Von Mises equivalent residual stress value in the center of the molten pool as a consequence of the process parameters. It can be noted that the stress value has a similar trend of variation across various process factors. With a constant laser power and a 200 mm/s increase in scanning speed, the maximum residual stress in the molten pool drops by approximately 21 MPa. With a 10 W increase in laser power and a constant scanning speed, the maximum residual stress in the molten pool falls by approximately 12 MPa.

**Figure 11:**
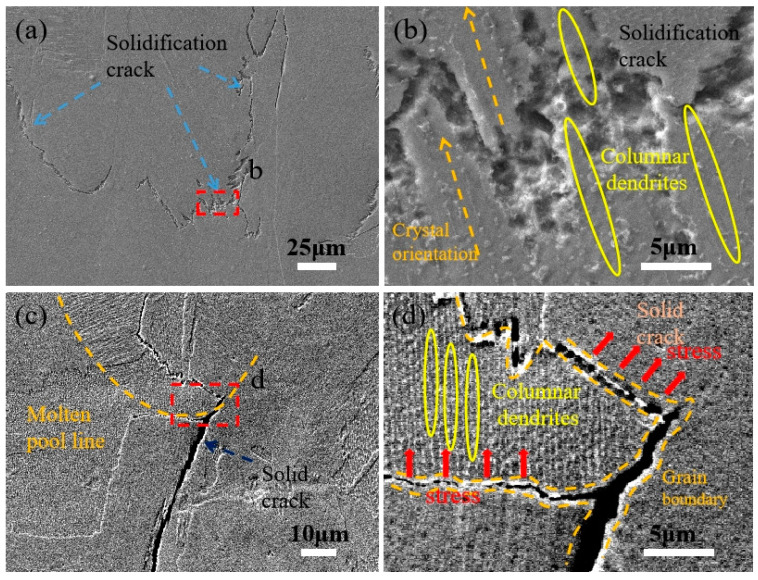
Crack morphology at high power, low scanning speed, and 90° interlaminar angle. (**a**) Solidification crack; (**b**) Local amplification of the area inside the dotted box in (**a**); (**c**) Solid crack; (**d**) Local amplification of (**c**).

Figure 11a depicts the fracture shape as a function of various process parameters. When the energy input per unit of time is high, the temperature differential in the molten pool is greater, and it is easier to build a developing columnar dendritic, which makes it harder for the molten metal to flow instantly and leaves gaps between the dendrites. Figure 10d demonstrates the simulation result that the higher the power, the higher the residual stress in the molten pool, and the easier it is to rip at the gap, leading to the creation of large-area solidification fissures, as depicted in Figure 11b. 

Figure 11c,d displays solid fractures with an interlaminar angle of 90°. The scanning traces of the molten pool are stacked parallel along the *z*-axis, one layer apart, at an interlaminar angle of 90°, which is more favorable to the epitaxial growth of the dendrites, and hence, generates a long grain boundary. Additionally, the crack will extend along the grain boundary. In the meantime, an increase in the power will cause an increase in stress, which causes cracks not only at the high-stress concentration area in the center of the molten pool but also along the grain boundary at the bottom of the molten pool.

### 3.4. Effect of Stress Distribution on Crack

**Figure 12:**
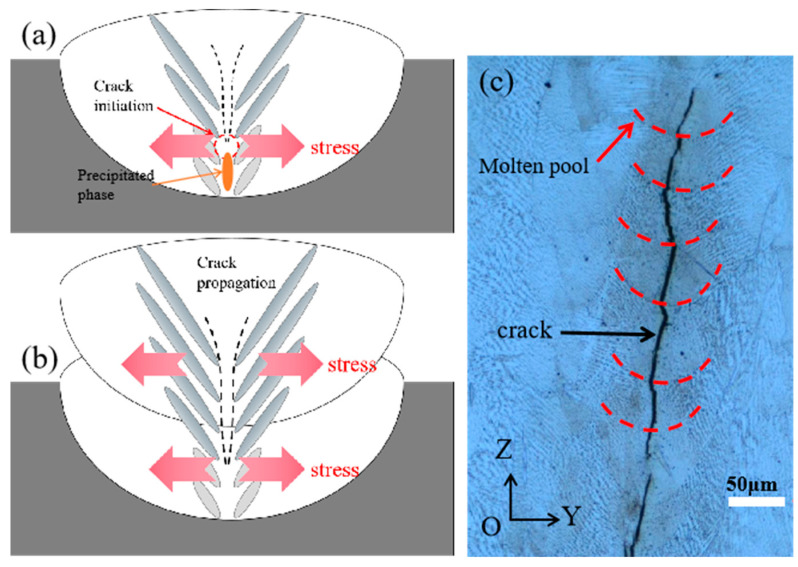
(**a**) Initiation of crack; (**b**) Propagation of crack; (**c**) Crack in experiment.

During the solidification process of a molten pool, the stress is predominantly distributed in the pool’s core region, corresponding with Mukherjee’s results [34]. Figure 12a depicts the beginning of a central crack. The accumulation of the precipitated phase at the grain boundary, which tears under the influence of stress and creates the initial fracture, is responsible for the initiation of cracks. In the layer-by-layer manufacturing process, as the building height grows, the dendrites continue to develop following the dendrite direction of the preceding layer and ultimately form columnar crystals [35,36,37]. In addition, the lengthy grain boundaries traverse the multilayered molten pool. Similar to the findings of other studies [9,38,39], the tension at the center of the molten pool increases along the building’s longitudinal axis. Since the molten pool is in the same building direction, the stress direction in the center of each molten pool layer is identical. As demonstrated in Figure 12b, the crack propagates along the grain boundary when the stress exceeds the fracture strength.

## 4. Conclusions

In this research, a three-dimensional finite element coupled thermo-mechanical model was constructed, the temperature field/stress field information, including the change of temperature gradient and equivalent stress at different locations of the molten pool, was analyzed quantitatively. From the evolution of the temperature and stress fields, the cause of grain boundary cracking at large angles was determined. Finally, the process parameters were optimized based on the finite element simulation and experimental results. The major findings of the study are as follows:

- During the solidification of the molten pool, the highest temperature gradients on the pool’s bottom and both sides reached 1.2×10^8^ K/m and 8×10^7^ K/m, respectively. The temperature distribution in the longitudinal portion of the molten pool caused the radiant distribution of stress. The stress value decreases slowly in the direction of scanning but rapidly in the direction perpendicular to scanning. The maximum stress of approximately 339 MPa.
- The stress concentration and precipitated phase enrichment in the middle of the molten pool of each layer caused crack initiation. The stress increased along the building direction. When the stress exceeded the fracture strength, the crack propagated along the grain boundary.
- For the case of process parameters with relatively high power or low scanning speed, the stress value of the molten pool during solidification was more than 370 MPa so as to form a large area of cracks. The adjustment of the rotation angle between the adjacent layers was effective at avoiding stress accumulation in the building direction and prevent the formation of long grain boundaries, thus avoiding crack propagation.

## Figures and Tables

**Table 1:** Chemical composition of FGH96 superalloy powder.

Element	Cr	Co	W	Mo	Ti	Al	Nb	C	Zr	Ni
wt%	15.5–16.5	12.5–13.5	3.8–4.2	3.8–4.2	3.55–3.9	1.95–2.3	0.60–0.80	0.045–0.060	0.03–0.06	bal.

**Table 2:** The used processing parameters in this study.

Laser Power (W)	Scanning Speed (mm/s)	Scanning Interval (μm)	Layer Thickness (μm)	Interlayer Angle (°)
260	800/1000/1200	80	40	67/90
270	800/1000/1200			67/90
280	800/1000/1200			67/90

## Data Availability

Data are contained within the article.
